# Play as a Method to Reduce Overweight and Obesity in Children: An RCT

**DOI:** 10.3390/ijerph17010346

**Published:** 2020-01-03

**Authors:** Antonio Manuel Sánchez-López, María José Menor-Rodríguez, Juan Carlos Sánchez-García, María José Aguilar-Cordero

**Affiliations:** 1 Department of Human Motricity and Sports Performance, University of Seville, 41004 Seville, Spain; antoniomsanchezlopez@gmail.com; 2 Research group CTS 367, Andalusia, Spain; jcsg750@gmail.com (J.C.S.-G.); mariajaguilar@telefonica.net (M.J.A.-C.); 3 Nursing Department, Ourense University Hospital Complex, 32005 Ourense, Spain; 4 Nursing Department, University of Granada, 18002 Granada, Spain

**Keywords:** obesity, play, physical activity, body composition, accelerometry

## Abstract

Background: Overweight and obesity are the result of a complex interaction between genetic and environmental factors, which begins prenatally. Aim: To analyse an intervention based on play as a means of improving the body composition of children who are overweight or obese. Methods: The Kids-Play study is a randomized clinical trial (RCT) consisting of 49 children aged 8–12 years on a nine-month intervention programme based on physical activity, play and nutritional advice. Controls had another 49 children, who received only nutritional advice. Results: The play-based intervention achieved a moderate-vigorous level of physical activity in the study group of 81.18 min per day, while the corresponding level for the control group was only 37.34 min. At the start of the intervention, the children in the study group had an average body fat content of 41.66%, a level that decreased to 38.85% by the end of the programme. Among the control group, body fat increased from 38.83% to 41.4% during the same period. Conclusions: The intervention programme considered, based on both play and nutritional recommendations, produced a decrease in body fat among children aged 8–12 years. However, the control group, which received only nutritional recommendations, experienced an increase in body weight.

## 1. Introduction

Today, obesity is considered a chronic disease, and many have termed it the epidemic of the twenty-first century. According to the World Health Organization (WHO), the body mass index (BMI) is the most useful measure of overweight and obesity, which are defined as BMI ≥ 25 and ≥30, respectively [1,2].

The incidence of overweight childhood and obesity has increased in recent decades. In Spain, according to the results of the enKid study [3], the prevalence of obesity among the population aged 2–24 years is approximately 12.4%. Spain has the fourth largest number of children with obesity problems in the EU [4], where approximately 22 million children are overweight [5].

Research has shown that overweight and obesity are the result of a complex interaction between genetic and environmental factors that begins prenatally [6]. Imbalance between caloric intake and energy expenditure is often the root cause of overweight and obesity. This imbalance can be aggravated by global dietary trends, with an increased consumption of energy-dense foods, high in fat and sugars but low in vitamins, minerals and other nutrients. Another factor is the decreasing level of physical activity and play, with the increasingly sedentary nature of school activities, as well as changes in transportation patterns and the development of technology-based games [7].

Obesity is associated with numerous diseases and metabolic, cardiovascular, respiratory, hormonal and psychological problems, among others. With overweight, it is the fifth leading risk factor for death in the world [8].

Obesity in children and adolescents is of particular significance, as this phenomenon is associated with increased morbidity and disability in adulthood. In this respect, school can be a favourable environment in which to guide the child’s behaviour toward a healthy lifestyle and the prevention of obesity and its associated diseases [9].

Various studies have shown that physical exercise based in play is an important component of weight loss programmes and, moreover, that it benefits the metabolic profile [10]. Other authors claim that weight loss is greater with a programme based on diet plus exercise, rather than diet or exercise alone [11].

However, the optimal amount of exercise needed to achieve long-term weight loss has not been established, and diverse recommendations have been made by health organisations. Thus, the Centers for Disease Control and Prevention and the American College of Sport Medicine both recommend a minimum of 30 min of physical activity of moderate intensity, most days of the week (i.e., 150 min/week) to improve health, while the Institute of Medicine suggests a minimum of 60 min/day of exercise and, equally, 150 min/week, to achieve the same degree of control of body weight [12,13].

The aim of this study is to analyse an intervention based on play as a means of improving the body composition of children with overweight or obesity.

## 2. Materials and Method

The Kids-Play study is a randomized clinical trial (RCT) registered at www.clinicaltrials.gov (identifier NCT02779647), was conducted in Granada (Spain) from June 2016 to February 2017.

Samples were collected in the paediatric outpatient clinics of 12 health centres and endocrinology clinics at the Granada Hospital Complex. The study universe was composed of 521 children with overweight or obesity who attended the paediatrics and endocrinology clinics. For a 95% confidence level of *p* = 0.5 and a maximum estimation error of 10% a sample size of *n* = 54 was required for each group. The presence of overweight and obesity is defined as a body mass index (BMI = weight/height^2^ (g/m^2^)) greater than the cutoff values established by the International Obesity Task Force for age and sex, in children and adolescents [14]. In addition, body composition was measured using bioelectrical impedance. The sample allocation was randomised, following a probabilistic technique, without replacement, whereby each child who arrived at the health centre and met the inclusion criteria was assigned a ticket bearing a serial number, by the researcher responsible for recruitment. All these tickets were placed in a large container, from which the principal investigator of the clinical trial extracted 54, which were assigned to the intervention group (IG). The following 54 numbers were assigned to the control group (CG). Inclusion Criteria: Obese children that want participate in a voluntary way in the research and sign the informed consent. Exclusion Criteria: Refused to participate, hormonal problems, age <8 or >12 years, orthopaedic problems, respiratory problems and other problems.

The intervention consisted of physical activity based on play, with four 90 min sessions per week for nine months (the school year). In which, both the subjects and the investigators were aware of the intervention. The physical activity was conducted by an expert in physical activity, at the school, in the afternoon, both during the week and on the weekend. In parallel, twice-monthly theoretical and practical sessions of nutritional advice were given to the children and their families. The study group performed the physical activity and received the nutritional advice, while the control group received only the theoretical and practical sessions on nutrition. The total of sessions was 144, the minimum number of sessions to consider valid that a child has completed the intervention was 115 (80%).

The physical activity consisted of sessions intended to be enjoyable and non-competitive. All sessions were structured in three parts: warming up, main activity and cooling down. In accordance with the needs of the study population, five minutes’ rest was usually allowed every half hour for hydration. The main activity consisted of popular games, sports suitable to the children’s needs, and alternative sports. In every case, the activities were mainly aerobic in nature, and care was taken to avoid excessive jumping. During the first few weeks, the sessions were controlled by the sports personnel, but later the children themselves chose the games they wanted to play. Thus, after a period during which different games and sports were learned, the children themselves decided which activities were most enjoyable, and this encouraged participation and compliance with the programme of physical activity.

The level of daily physical activity was assessed using ActiGraph wGT3X-BT (ActiGraph LLC, Pensacola, FL, USA) accelerometers, which were worn on the right hip by all children in the study population for seven days, except during the hours of sleep. In this way it was possible to objectively analyse whether the study group met the WHO recommended levels of activity for their age group, and to determine the differences between cases and controls.

The children’s body composition was measured before and after the intervention, by bioelectrical impedance and using the InBody 720 (Biospace Co. Ltd., Seoul, Korea) body composition analyser [15,16]. The human body is composed of water, protein, body fat and minerals, and all of these components can be quantified by bioelectrical impedance. It is essential to measure these variables in order to determine the effect of physical activity on the children in the study group, compared to the controls.

Before starting this research, an initial project was presented for approval by the Research Ethics Committee of the province of Granada (Granada CIS), Spain.

The welfare and privacy of those participating in any research study is the responsibility of the researchers concerned. It is explicitly stated that this study complies with the ethical rules proposed by the Committee on Research and Clinical Trials, as set out in the 1964 Declaration of Helsinki (revised in Fortaleza, Brazil, 2013).

A descriptive analysis was performed of the main study variables. Quantitative variables are described by the mean, the standard deviation, the median and the percentiles, and qualitative ones by percentages. The effectiveness of the intervention and the change in the variables (such as weight, height and BMI) and in the quality of life were tested by the Student *t* test or the Wilcoxon test for related samples, according to the normality or otherwise of the distribution of the variables. The association between qualitative variables was examined by the Pearson chi-square test or Fisher’s exact test. The level of significance assumed was *p* < 0.05. To analyze the change in time of body composition values per group, a repeated measures ANOVA has been used, time is considered as an intra-subject factor, the group as an inter-subject factor, and the interaction between both. All data were analysed using SPSS v.19 statistical software (IBM, Armonk, NY, USA).

## 3. Results

**Figure 1:**
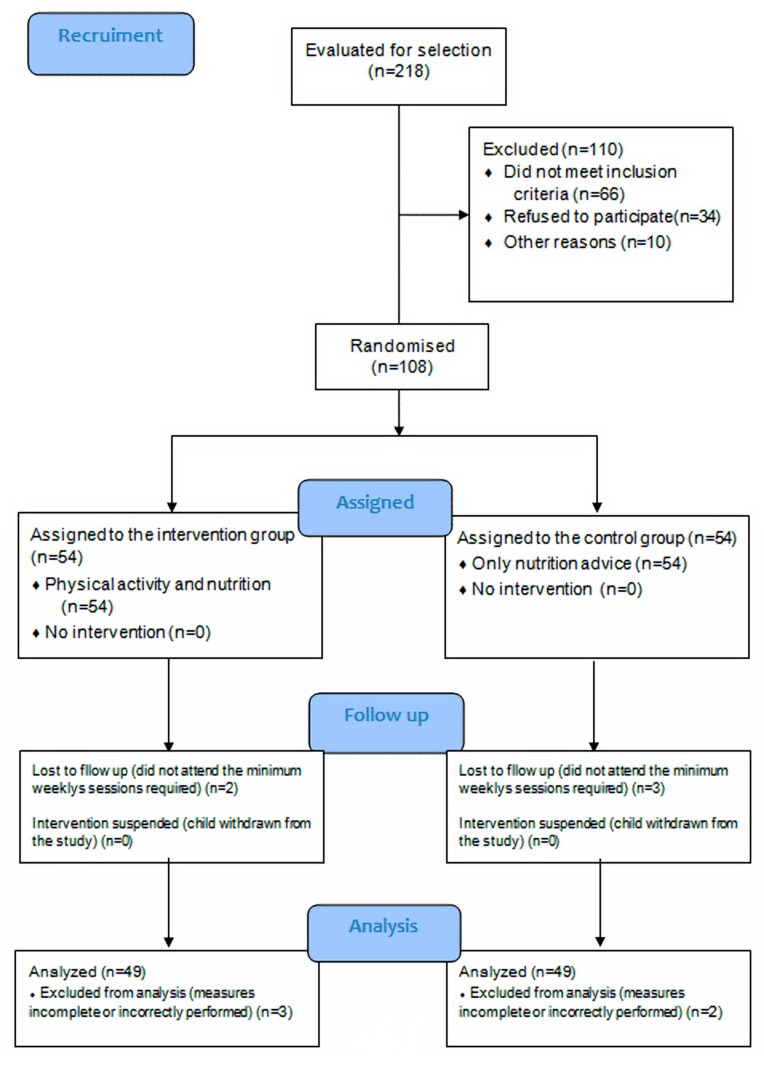
Selection of the study sample.

The flow chart in Figure 1 shows the process applied in selecting the study sample. Out of the 108 people who initially met the inclusion criteria, five participants dropped out of the programme claiming a lack of interest in the performance of physical exercise and did not complete the intervention. Data from five people were not included in the final analysis, because of not having attended 80% of the scheduled sessions because of health problems. Therefore, adherence to the programme stood at 90.8% (98 out of 108) and 90.74% in each group.

The study population was composed of 52 boys and 46 girls, distributed as intervention (49) and controls (49). The average age of the children was 10.65 ± 1.38 years, the average weight was 66.05 kg and the average height was 150.75 cm, equivalent to an average BMI of 28.60. Table 1 shows the baseline characteristics of the sample by groups.

Table 2 shows that the level of physical activity, as measured by the accelerometer, was significantly higher in the study group than in the controls, after a typical intervention week. The average quantity of moderate-vigorous physical activity (MVPA) in the study group was 81.18 min/day, compared to 37.34 min/day by the controls. All the children, both those in the study group and the controls, were more active at the weekend (MVPA.WEd) than on weekdays (MVPA.Wd).

The WHO recommends 60 min of MVPA per day. In our analysis, MVPA is taken as a qualitative variable (recommended > 60), evaluating the percentage of children in each group who met the WHO recommendation.

Table 2 show that 100% of the intervention group achieved the recommended level of MVPA, compared with 4.4% of the controls (chi-square test, *p* < 0.001). In both groups, the number of children who met the physical activity recommendations differed between weekdays and the weekend.

Table 2 reflects the significant difference between the average of 13,395 steps a day (Steps Total) taken by the children in the study group and the 8601 by those in the control group. According to Adams et al., an average daily value of 11,500 steps is recommended [17]. From the data shown in Table 2, it can be seen that 93.87% of the study group met this recommendation, compared with 4.4% of the control group (chi-square test, *p* < 0.001). The data also show that the children took a greater number of steps per day at the weekends (StepsWEd) than on weekdays (StepsWd).

Table 3 describes the changes in the main variables for body composition, reflecting the differences between the study group and the control group. The difference between the “pre” and “post” values for the percentage of fat was significant in both groups (*p* < 0.001). In the study group, the children had an initial average fat percentage of 41.66%, and this value decreased to 38.85% by the end of the study. However, in the group that received only nutritional advice, the average percentage of body fat actually rose, from 38.83% to 41.4%. There was also a significant difference between the groups as regards the weight the children needed to lose in order to reach their recommended weight according bioelectrical impedance. A similar pattern was observed with the BMI, as shown in the same table.

The children in the study group presented a higher degree of obesity at the outset than the control group. In the “post” values, the difference in BMI was small, with 28.02 and 28.92 respectively, but both results are significant.

Table 3 shows the changes in body composition, for the study group and the controls. The largest difference between the groups was recorded for body fat. Among the children who took part in the physical activity programme and also received nutritional advice, body fat levels decreased from 28.90 to 26.56 kg, while those in the control group, with no added physical activity, experienced an increase in body fat, from 26.83 to 29.84 kg. This finding highlights the importance of physical activity and play in reducing excess body fat.

## 4. Discussion

Based on a review of current literature in this field, the Kids-Play study aims to incorporate the best features of previous research, to propose an effective educational programme for children with overweight or obesity, achieving lasting weight loss [18].

Children who are overweight often lack motivation at school and in sports in general. Moreover, they often suffer rejection by their peers, due to their lack of skills at physical activity [19]. Taking into account these considerations, we designed a play-based and non-competitive educational intervention, as a motivating practice for this population. This intervention lasted a complete school year, in order to avoid the rebound effect (a programme duration of at least 10 months has been recommended) [20,21]. The children’s families were encouraged to participate in this intervention, and attended monthly educational sessions at which advice was given on good nutrition and healthy habits. This approach is in line with the recommendations of Gunnarsdottir et al. (2011), according to which the parents’ contribution is of fundamental importance in motivating their children and thus improving their health status and reducing overweight and obesity [22].

The results obtained show that an educational intervention based on play and on nutritional advice effectively reduces overweight and obesity in children [1]. These results are comparable to those obtained by Danielsen et al. (2013) and Walther et al. (2009) [23,24]. In contrast, Wallman et al. (2009) failed to reduce overweight with an intervention based on aerobic exercise and nutritional advice. However, the duration of this programme was only eight weeks, which is a very short time to achieve meaningful results [7]. On the other hand, Willis et al. (2012) reported that aerobic exercise is the best method to reduce overweight and fat mass, as shown in our own intervention [25].

The level of physical activity achieved by the study group exceeded the WHO recommendations of at least 60 min per day of moderate or vigorous physical activity [26]. In fact, the study group recorded an average of 81.18 min per day with this type of activity. In contrast, the children in the control group (N) only spent 37.34 min practicing moderate or vigorous physical activity. This is a key difference, which accounts for the decrease in the percentage of fat and in the BMI in the study group and for the increased values in the control group. The same conclusion was drawn by Fisher et al. (2011), who measured a significant association between BMI and the level of moderate or vigorous physical activity by children aged 8–10 years [27].

Accelerometry has been shown to be an effective and objective method to assess the level of physical activity of children with overweight and obesity. Seven days is the minimum time recommended in which to reliably measure the subjects’ level of physical activity. In this respect, Ojiambo et al. (2011) applied a period of 7.4–8.5 days in order to include the physical activity performed both on weekdays and at the weekend [28].

In our own study, physical activity was greater at the weekends than during the week, both for the study group and for the controls. However, Blaes et al. (2011) studied 361 children and reported that their physical activity was greater on the days when they attended school [29]. A similar conclusion was drawn by Kawahara et al. (2011) [30].

The analysis of body composition by bioelectrical impedance is a reliable way to determine overweight and obesity in children. This assessment is shared by Kabiri et al. (2016) in their study of the reliability of bioelectrical impedance in children, in which excellent test-retest reliability and high specificity for the classification of fat and obesity was demonstrated. This study compared bioelectrical impedance with bone densitometry scanning (DEXA), and found both methods to be highly reliable for analysing the body composition of children [31]. Meredith-Jones et al. (2015) also found bioelectrical impedance to be a reliable method for this analysis [32].

In our study, bioelectrical impedance showed that the study group achieved an improved body composition, while that of the control group worsened. The main difference observed was in the fat content, which increased in the children who did not take part in added physical activity, in contrast to the study group. Lee et al. (2015) also found that physical activity is the main variable in improving body composition [33]. Moreover, Smith et al. (2016) support the theory that aerobic endurance should be increased via play as a means of reducing overweight and obesity in children, and enabling them to achieve an appropriate body composition [34].

## 5. Conclusions

The main conclusion drawn from the Kids-Play study is that a nine-month intervention programme based on play and nutritional recommendations led to decreased BMI and body fat in children aged 8–12 years. However, the group that only received nutritional recommendations gained weight.

Involvement of family members is important to motivate the children and to establish healthy habits and thus a healthy future. The workshops in which the parents took part highlighted the problems associated with overweight and obesity. However, most parents failed to understand that overweight is an alteration that causes serious health problems, not only in the future but also in the present. This long-term commitment is necessary in order to avoid the rebound effect.

Accelerometry is a reliable and effective way to assess the level of children’s physical activity. It is important that children should achieve a level of moderate or vigorous physical activity of 60 min a day, at least, on average, in order to achieve significant results in reducing overweight and obesity. In this respect, a quantity of 11,500 steps taken each day is sufficient to produce significant changes in body composition.

The children in both groups were more physically active at the weekend than during the week, possibly because this is when they have more free time for such activities. In contrast, during the week the children are often busy with their studies and extracurricular activities, which makes regular physical activity more difficult.

To sum up the conclusions drawn from this study, in order to avoid the rebound effect, children’s habits must be changed. To do so, their level of physical activity and play, as part of everyday activities, should be increased, both at school, and with the family and friends.

## Figures and Tables

**Table 1:** Baseline demographic and clinical characteristics for each group.

Characteristic	Intervention Group (*n* = 49)	Control Group (*n* = 49)	*p*
	Mean (SD)	Mean (SD)	
Age	10.39 (1.28)	10.47 (1.43)	0.767
Weight (kg)	66.70 (15.58)	64.51 (16.63)	0.502
Height (cm)	148.61 (9.96)	151.69 (11.87)	0.167
BMI	29.29 (3.83)	27.26 (3.62)	0.008
Fat (%)	41.91 (6.24)	38.63 (5.82)	0.009
Water (kg)	27.72 (6.16)	27.43 (6.11)	0.819
Protein (kg)	7.22 (1.69)	7.37 (1.76)	0.667
Mineral (kg)	2.67 (0.66)	2.75 (0.69)	0.567
Fat (kg)	28.04 (8.95)	26.34 (9.45)	0.363
Muscle (kg)	20.01 (4.97)	20.62 (5.48)	0.570

**Table 2:** Difference of the means for Moderate-Vigorous Physical Activity (MVPA) and steps between the groups.

Moderate–Vigorous Physical Activity (MVPA)					
Physical Activity	Intervention Group (*n* = 49)		Control Group (*n* = 49)		
	Mean (SD)	% >60 min/day	Mean (SD)	% >60 min/day	*p*
MVPA/Day weekdays	76.14 (21.43)	77.55% (38)	36.44 (10.55)	8.16% (4)	<0.001
MVPA/Day weekend	91.82 (23.75)	95.91% (47)	39.45 (14.36)	12.24% (6)	<0.001
MVPA/Day Total	81.18 (13.42)	100% (49)	37.34 (10.03)	4.4% (2)	<0.001
**Steps**					
**Steps**	**Intervention Group (*n* = 49)**		**Control Group (*n* = 49)**		
	**Mean (SD)**	**% >11,500 steps/day**	**Mean (SD)**	**% >11,500 steps/day**	***p***
Steps/Day weekdays	12,879 (2817)	77.55% (38)	8410 (1763)	4.4% (2)	<0.001
Steps/Day weekend	14,656 (3322)	87.76% (43)	9107 (2609)	12.24% (6)	<0.001
Steps/Day Total	13,395 (1796)	93.87 (46)	8601 (1622)	4.4% (2)	<0.001

**Table 3:** Body composition “pre” and “post” intervention, by groups.

Body Composition	Group	Pre Intervention	Post Intervention	Group *p*	Time *p*
		Mean (SD)	Mean (SD)		
Weight (kg)	IG	67.24 (17.65)	66.81 (16.72)	0.001	0.001
	CG	64.94 (17.38)	70.57 (16.22)		
BMI	IG	29.73 (4.12)	28.08 (4.16)	0.001	0.001
	CG	27.54 (3.75)	28.91 (3.25)		
Fat %	IG	41.66 (5.39)	38.82 (6.69)	0.001	0.001
	CG	38.82 (5.82)	41.39 (4.91)		
Fat (kg)	IG	28.89 (9.54)	26.55 (9.29)	0.001	0.001
	CG	26.82 (9.53)	29.93 (9.19)		
Water (kg)	IG	28.30 (6.72)	29.50 (7.13)	0.466	0.001
	CG	27.77 (6.14)	29.27 (5.73)		
Protein (kg)	IG	7.38 (1.80)	7.84 (1.83)	0.284	0.001
	CG	7.48 (1.77)	8.10 (1.51)		
Mineral (kg)	IG	2.71 (0.69)	2.89 (0.67)	0.001	0.001
	CG	2.78 (0.68)	3.17 (0.59)		
Muscle (kg)	IG	20.49 (5.32)	21.70 (5.50)	0.547	0.001
	CG	20.88 (5.45)	21.90 (5.08)		

IG: Intervention group, CG: Control Group, BMI: Body Mass Index, SD: Standard Deviation.

## Clinical Trial Registration

This trial has been registered at www.clinicaltrials.gov (identifier NCT02779647).
